# Oxidative stress, mitochondrial damage, and cores in muscle from calsequestrin-1 knockout mice

**DOI:** 10.1186/s13395-015-0035-9

**Published:** 2015-04-18

**Authors:** Cecilia Paolini, Marco Quarta, Lan Wei-LaPierre, Antonio Michelucci, Alessandra Nori, Carlo Reggiani, Robert T Dirksen, Feliciano Protasi

**Affiliations:** CeSI - Center for Research on Ageing & DNICS - Department of Neuroscience, Imaging and Clinical Sciences, University G. d’Annunzio, Via L. Polacchi, 11, I-66013 Chieti, Italy; Department of Biomedical Sciences, University of Padova, Via U. Bassi 58/B, I-35131 Padova, Italy; Department of Pharmacology and Physiology, University of Rochester Medical Center, 601 Elmwood Ave., Rochester, NY 14642 USA; Department of Neurology and Neurological Sciences, Stanford University, 450 Serra Mall, Stanford, CA 94305 USA

**Keywords:** Excitation-contraction (EC) coupling, Mitochondria, Ryanodine receptor (RYR)

## Abstract

**Background:**

Mutations in the gene encoding ryanodine receptor type-1 (RYR1), the calcium ion (Ca^*2*+^) release channel in the sarcoplasmic reticulum (SR) of skeletal muscle, are linked to central core disease (CCD) and malignant hyperthermia (MH) susceptibility. We recently reported that mice lacking the skeletal isoform of calsequestrin (CASQ1-null), the primary Ca^*2*+^ buffer in the SR of skeletal muscle and a modulator of RYR1 activity, exhibit lethal heat- and anesthetic-induced hypermetabolic episodes that resemble MH events in humans.

**Methods:**

We compared ultrastructure, oxidative status, and contractile function in skeletal fibers of *extensor digitorum longus* (*EDL*) muscles in wild type (WT) and CASQ1-null mice at different ages (from 4 to 27 months) using structural, biochemical, and functional assays.

**Results:**

About 25% of fibers in *EDL* muscles from CASQ1-null mice of 14 to 27 months of age exhibited large areas of structural disarray (named *core-like regions*), which were rarely observed in muscle from age-matched WT mice. To determine early events that may lead to the formation of cores, we analyzed EDL muscles from adult mice: at 4 to 6 months of age, CASQ1-null mice (compared to WT) displayed significantly reduced grip strength (40 ± 1 *vs*. 86 ± 1 mN/gr) and exhibited an increase in the percentage of damaged mitochondria (15.1% *vs*. 2.6%) and a decrease in average cross-sectional fiber area (approximately 37%) in EDL fibers. Finally, oxidative stress was also significantly increased (25% reduction in ratio between reduced and oxidized glutathione, or GSH/GSSG, and 35% increase in production of mitochondrial superoxide flashes). Providing *ad libitum* access to N-acetylcysteine in the drinking water for 2 months normalized GSH/GSSG ratio, reduced mitochondrial damage (down to 8.9%), and improved grip strength (from 46 ± 3 to 59 ± 2 mN/gr) in CASQ1-null mice.

**Conclusions:**

Our findings: 1) demonstrate that ablation of CASQ1 leads to enhanced oxidative stress, mitochondrial damage, and the formation of structural cores in skeletal muscle; 2) provide new insights in the pathogenic mechanisms that lead to damage/disappearance of mitochondria in cores; and 3) suggest that antioxidants may provide some therapeutic benefit in reducing mitochondrial damage, limiting the development of cores, and improving muscle function.

**Electronic supplementary material:**

The online version of this article (doi:10.1186/s13395-015-0035-9) contains supplementary material, which is available to authorized users.

## Background

In skeletal muscle, excitation-contraction (EC) coupling is a process by which depolarization of the transverse (T)-tubule membrane is mechanically linked to the release of calcium ions (Ca^2+^) from the sarcoplasmic reticulum (SR) and, hence, activation of muscle contraction [1]. EC coupling in skeletal muscle involves direct, bi-directional communication between T-tubule voltage sensors (dihydropyridine receptors, α_1s_DHPRs) and SR Ca^2+^ release channels (ryanodine receptor type-1, RYR1) [2,3]. Several other junctional proteins, including calsequestrin-1, triadin, junctin, JP-45, stac3, and FK-506 binding protein, interact with RYR1 and α_1_sDHPR to form a macromolecular machinery that controls Ca^2+^ release [4]. Several pathological conditions that adversely affect striated muscle function are associated with mutations in EC coupling proteins that result in alterations in the proper control of SR Ca^2+^ release [5].

Mutations in the RYR1 gene account for the majority of cases of malignant hyperthermia susceptibility (MHS) and central core disease (CCD) in humans [6-8]. CCD is one of the most common congenital myopathies: CCD patients exhibit fetal hypotonia, proximal muscle weakness, and a significant delay in motor development [9,10]. Diagnosis of CCD is confirmed by histological examination of muscle biopsies showing amorphous central areas (or cores, typically found in type I fibers) lacking glycolytic/oxidative enzymes and mitochondria [11]. Disorganization of the contractile elements and sarco-tubular systems is typically observed within the core regions [12]. MHS, on the other hand, is a pharmacogenetic disorder characterized by life-threatening episodes triggered by volatile anesthetics (for example, halothane, isoflurane, etc.) and depolarizing muscle relaxants (for example, succinylcholine) [13-15] that occurs in the absence of an overt myopathy. CCD and MHS are often related: some patients with CCD test positive for MHS [16,10] and, thus, are considered at risk for malignant hyperthermia (MH) [17].

Calsequestrin (CASQ) is a highly acidic protein that binds Ca^2+^ that is concentrated at the junctional face of the terminal cisternae, near the sites of RYR1 Ca^2+^ release [18-20]. There are two isoforms of mammalian CASQ that are products of different genes: the skeletal (CASQ1) and cardiac (CASQ2) isoforms, which are expressed at different levels during development of fast- and slow-twitch skeletal muscle fibers [21,22]. In addition to being important for SR Ca^2+^ binding/storage, CASQ1 also modulates the RYR1 Ca^2+^ release channel activity [23,24].

Our structural and functional studies in CASQ1-knockout (or null) mice revealed that while CASQ1 ablation is not lethal [25], skeletal muscle from CASQ1-null mice exhibits significant structural remodeling of Ca^2+^ release units (CRUs) and impaired Ca^2+^ handling [25-27]. Abnormal CRUs (that is, forming multiple elements with reduced junctional SR lumen) exhibit rapid and severe SR Ca^2+^ depletion in response to repetitive stimulation [28]. As a result, EC coupling is unable to support prolonged Ca^2+^ transients required to sustain force production during tetanic stimulation [26,28]. Importantly, CASQ1-null mice also exhibit life-threatening, hypermetabolic episodes characterized by sustained muscle contractures, rhabdomyolysis, and uncontrolled elevations in core body temperature when exposed to either halothane or environmental heat stress [29-31], which closely resemble human MHS [14,15]. However, mutations in the CASQ1 gene have not been identified in MH patients. In fact, Kraeva *et al*. [32] failed to identify any mutations within the CASQ1 coding region in 75 unrelated MHS patients diagnosed by caffeine-halothane contracture test, thus concluding that CASQ1 is an unlikely genetic locus for MHS within the North American population [33]. A similar conclusion was reached in another study of European MHS patients (Vincenzo Sorrentino, University of Siena, personal communication). However, a missense mutation in the CASQ1 gene (N244G, the first CASQ1 disease mutation identified in humans) was recently reported in a group of patients with a myopathy characterized by weakness, fatigue, and the presence of large vacuoles containing characteristic inclusions resulting from the aggregation of SR proteins [34]. The MH status of individuals that possess the N244G mutation in CASQ1 is currently unknown.

Since CASQ1 exhibits an MHS-like phenotype and MHS occurs in some CCD patients, we hypothesized that CASQ1-null mice may develop a myopathy resembling CCD. To test this hypothesis, we compared skeletal muscle ultrastructure, oxidative status, and contractile function in wild type (WT) and CASQ1-null mice at different ages (4 to 6, 14, 20, 24, and 27 months). Our results indicate that CASQ1-null mice exhibit impaired muscle function (that is, reduced force output), mitochondrial damage, and structural core-like regions similar to that described previously in RYR1 mouse models of MH/CCD [35,36]. We found that increased oxidative stress appears to be a key trigger for this myopathic phenotype in CASQ1-null mice as treatment with the antioxidant N-acetylcysteine (NAC) provides significant protection from the development of structural and functional deficits.

## Methods

All functional, structural, and molecular analyses were carried out in either a) *extensor digitorum longus* (EDL; predominantly fast-twitch fibers), the muscle group which is more affected by CASQ1 ablation [25,26], or b) *flexor digitorum brevis* (FDB; approximately 80% of fast-twitch fibers; [37]) for single cell experiments.

### Ethics statement

All experiments were conducted according to the Directive of the European Union 2010/63/UE and the National Institutes of Health *Guide for the Care and Use of Laboratory Animals*. All animal protocols were approved by the Committee on the Ethics of Animal Experiments of the University of Chieti (CEISA; Permit Number: 40) and the University Committee on Animal Resources at the University of Rochester Medical Center. All surgeries were made to minimize animal suffering: animals were anesthetized and then sacrificed by cervical dislocation.

### CASQ1-null mice

CASQ1-null mice [25] were housed in micro-isolator cages, at 20°C in a 12-h light/dark cycle, with free access to water and food. As life expectancy of CASQ1-null male mice is significantly reduced (see Additional file 1: Figure S1; see also refs. [29,31]), all experiments conducted in this study were performed using female WT and CASQ1-null animals (due to shortage of aged male CASQ1-null mice). For *in vivo* antioxidant treatment, mice were provided *ad libitum* access to drinking water containing NAC (1% *w*/*v*).

### Grip strength test

Grip strength of WT and CASQ1-null mice from three different age groups (4 to 6, 14, and 22 to 24 months of age) was measured as described by Connolly *et al*. [38]. Briefly, mice were held by the tail and lowered to a metal grating connected to the shaft of a Shimpo Fgv 0.5X force transducer (Metrotec Group, San Sebastián, Spain). Once the mouse had firmly grabbed the grating, a gentle pull was exerted on the tail. Measurements of peak force generated by each mouse using fore and hind limbs were repeated three times with appropriate intervals (at least 30 s) to avoid fatigue. Average peak force values were normalized to total body mass measured immediately before each experiment.

### Force and contraction kinetics of isolated EDL muscles

EDL muscles were dissected from the hind limb of WT and CASQ1-null mice of three different age groups (4 to 6, 14, and 22 to 24 months old). Force and contraction kinetics were measured as described by Paolini *et al*. [25]. Excised muscles were continuously perfused with oxygenated Krebs solution (NaCl 118 mM, KCl 4.7 mM, MgSO_4_ 1.2 mM, KH_2_PO_4_ 1.1 mM, glucose 11.1 mM, CaCl_2_ 2.5 mM, NaHCO_3_ 25 mM, pH 7.4) and mounted between a force transducer (AME-801 SensorOne, Sausalito, CA, USA) and micromanipulator-controlled shaft in a small chamber. The temperature of the chamber was maintained at 25°C. The stimulation conditions were optimized, and muscle length was increased until force development during tetanus was maximal. Single twitches and fused tetani (120 Hz, 0.5-s duration) under isometric conditions were recorded with rest intervals suitable to avoid fatigue. Specific force (mN/mm^2^) and time to peak contraction (s) were measured.

### Preparation of samples for histology and EM

EDL muscles were carefully dissected from WT and CASQ1-null mice at different ages (4 to 6, 14, 20, 24, and 27 months). Muscles were fixed at room temperature (RT) in 3.5% glutaraldehyde in 0.1 M sodium cacodylate buffer, pH 7.2, for 2 h and kept in fixative until further use. Small bundles of fibers were then post-fixed and embedded as described by Paolini *et al*. [25]. For histological analyses, longitudinal and cross-oriented semithin sections (250 nm) were cut with a Leica Ultracut R microtome (Leica Microsystem, Vienna, Austria) using a Diatome diamond knife (Diatome Ltd. CH-2501 Biel, Switzerland). After staining with toluidine blue dye, the sections were viewed on a Leica DMLB fluorescence microscope (Leica Microsystem, Vienna, Austria). For electron microscopy (EM), ultrathin sections were cut (approximately 50 nm), stained in 4% uranyl acetate and lead citrate, and examined with a Morgagni Series 268D electron microscope (FEI Company, Brno, Czech Republic) equipped with a Megaview III digital camera.

### Quantitative analyses of histological and EM specimens

1) Measurements of fiber cross-sectional area (CSA) were performed on cross-oriented semithin sections of EDL muscles from WT and CASQ1-null mice of 4 to 6, 14, and 24 months of age (by histological analysis). Images were selected and CSA calculated using the AnalySIS software (Olympus Soft Imaging Solutions GmbH, Munster, Germany). 2) Mitochondrial volume was determined in adult (4 to 6 months) and aged (27 months) WT and CASQ1-null mice before and after NAC treatment. Mitochondrial volume was calculated using the well-established stereology point-counting technique [39,40] in EM micrographs taken at 14,000× of magnification after superimposing an orthogonal array of dots at a spacing of 0.20 μm to the electron micrographs. The ratio between the number of dots falling within the mitochondrial profile and the total number of dots covering the whole image was used to calculate the relative volume of the fiber occupied by mitochondria. 3) The number of altered mitochondria was determined in EDL muscles from 4- to 6-month-old WT and CASQ1-null mice (in the absence or presence of NAC treatment) using the same set of EM micrographs as described for the mitochondrial volume measurements. Mitochondria with any one (or more) of the following ultrastructural alterations were classified as damaged: a) swollen mitochondria, b) mitochondria with clear disruption of the external membrane and/or internal cristae, c) mitochondria containing vacuoles, and d) mitochondria containing myelin figures. The quantification was reported as the percentage of all mitochondria evaluated in the fiber. 4) Determination of the number of damaged fibers in adult (4-month-old) and aged (four time points between 14 and 27 months of age) mice was performed on histological sections stained with toluidine blue. Quantitative analysis was performed at multiple time points for each genotype. Individual fibers were visually scored for the presence of *unstructured cores* and *contracture cores*, as described in Boncompagni *et al*. 2009 [35]. The number of fibers with alterations was presented as a percentage of all fibers evaluated.

### Immunohistochemistry

Dissected EDL muscles from 27-month-old WT and CASQ1-null mice were fixed in 2% paraformaldehyde in phosphate-buffered saline (PBS) for 20 min at RT. Small bundles of fibers were blocked for 1 h in PBS containing 1% bovine serum albumin (BSA), 10% goat serum, and 0.5% TRITON X-100 and then incubated overnight at 4°C with primary antibodies. After washing in PBS/BSA, fibers were incubated for 1 h at RT with secondary antibodies, washed again, and finally mounted with an anti-bleach medium. Code, specificity, working dilution, and the sources of primary and secondary antibodies used were as follows: mouse anti-RYR 34C, 1:30 [41] (Developmental Studies Hybridoma Bank, The University of Iowa), and Cy3 goat anti-mouse, 1:200 (Jackson ImmunoResearch Laboratories, West Grove, PA, USA). The specimens were viewed using a laser-scanning confocal microscope interfaced with an inverted Zeiss Axiovert microscope (LSM510 META, Zeiss, Germany).

### Gene profile analyses

Microarray analyses were performed by CRIBI - Biotechnology Center and Biology Department Padua University - using spotted oligonucleotide Micro-CRIBI Mouse Oligo 13.4k Array V_0 (Operon Version 1.1), GEO accession platform number GPL6747. This microarray set consists of 13.440 70-mer oligonucleotides designed on Mouse Unigene clusters, mainly in the 3′ end terminal region. Briefly, each oligonucleotide is spotted by a robotic station Biorobotics Microgrid II, in one replicate on a MICROMAX glass slide SuperChip I (Cat No MPS696) provided by PerkinElmer Life Sciences Inc. The deposition is qualitatively assessed by a scan of the spotted slide at 10-μm resolution, using a ScanArray LITE (PerkinElmer, Waltham, MA, USA). Code GSE25984 at: http://www.ncbi.nlm.nih.gov/geo/query/acc.cgi?token=xdojfeeesguuwji&acc=GSE25984.

For all genes, *P* = 0.05, −0,65 > LogR > 0,65.

### Western blot analysis

Preparation of total homogenates from EDL muscles of 4-month-old female WT and CASQ1-null mice were performed as previously described [25]. Identification of peroxisome proliferator-activated receptor gamma coactivator 1-alpha (PGC-1α) was obtained in three different EDL muscles from three different animals using a polyclonal antibody (H-300 Santa Cruz Biotechnology, Dallas, TX, USA) diluted at 1:200. Immuno-detection was performed using an anti-rabbit alkaline phosphatase-conjugated antibody at a 1:10.000 dilution. For densitometric analysis, scans of nitrocellulose membranes were quantified using Scion Image J Software (National Institutes of Health, Bethesda, MD, USA). Normalization to actin was performed using Ponceau S red staining to assess total protein loading (100 μg per lane). Mean optical density (O.D.) values of PGC-1α bands were normalized to that observed for WT muscles, set as 100 O.D., and data were presented as density ratio. As the PGC-1α antibody detected a doublet in all samples, the two bands were compared with that of a positive control (A-673 nuclear extract from rhabdomyosarcoma cells, Santa Cruz Biotechnology, Dallas, TX, USA) to determine the band corresponding to PGC-1α: PGC-1α was identified as the lower band in the doublet observed upon Western blot analysis (see Additional file 2: Figure S2).

### *In vivo* electroporation of mt-cpYFP cDNA into hind limb footpads of anesthetized mice

WT and CASQ1-null mice (4 to 5 months old) were anesthetized by intraperitoneal injection of 100 mg/kg ketamine, 10 mg/kg xylazine, and 3 mg/kg acepromazine. Hind limb footpads of anesthetized mice were then injected subcutaneously with bovine hyaluronidase (7 μl/foot, 2 μg/μl). One hour later, hind limb footpads were subcutaneously injected with 30 μg of mitochondria-targeted circularly permuted yellow fluorescent protein (mt-cpYFP) cDNA (total volume 10 μl in 71 mM NaCl) using a 30-gauge needle. The footpad was then electroporated (100 V/cm, 20-ms duration, 20 pulses delivered at 1 Hz) using subcutaneous gold-plated electrodes placed perpendicular to the long axis of the muscle. Individual FDB fibers were isolated by enzymatic dissociation 1 week after electroporation as described previously [42].

### mSOF measurement and analyses

Acutely isolated FDB fibers (from *n* = 4 to 5 mice) were plated on glass-bottom dishes in a rodent Ringer’s solution (146 mM NaCl, 5 mM KCl, 1 mM MgCl, 2 mM CaCl_2_, 10 mM HEPES, pH 7.4) and then loaded with 10 nM tetramethylrhodamine ethyl ester (TMRE) to enable simultaneous monitoring of changes in mitochondrial membrane potential during mitoflash activity. Mt-cpYFP and TMRE were excited using 488-nm (8× attenuation) and 543-nm (64× attenuation) lasers and detected at 515/30-nm and 605/75-nm emission, respectively. Time-lapse *x*,*y* images were acquired at 1.24 s/frame and 512 × 512 resolution, with a total of 100 frames, using a Nikon Eclipse C1 Plus Confocal microscope equipped with a SuperFluor 60x (1.4 NA) oil-immersion objective. Automated detection and analysis of individual mitochondrial superoxide flash (mSOF) events during time-lapse *x*,*y* imaging was performed using ‘Flash Collector,’ a MATLAB-based image analysis program described in detail previously [42]. Flash frequency, amplitude, and full duration at half maximal amplitude (FDHM) were expressed as the number of events/1,000 μm^2^∙100 s, Δ*F*/*F*_0_, and seconds, respectively. Output data from Flash Collector were tabulated, averaged, and evaluated for statistical significance using Microsoft Excel and SigmaPlot software suites.

### Glutathione assay

Control and NAC-treated (for 2 months) 4-month-old WT and CASQ1-null mice were sacrificed and hind limb muscles removed. Hind limb muscles were homogenized, and total glutathione and oxidized glutathione (GSSG) levels were measured according to [43]. Briefly, 0.1 g of tissue sample was homogenized in 1 ml of fresh extraction buffer (0.1% Triton X-100 and 0.6% sulfosalicylic acid in 0.1 M potassium phosphate buffer with 5 mM EDTA disodium salt, pH 7.5) on ice three times using an MICCRA D-1 Homogenizer (ART Prozess- & Labortechnik GmbH & Co., Müllheim, Germany). After homogenization, 5% trichloroacetic acid was added to the solution, and the homogenized tissue sample was centrifuged at 8,000 × *g* for 10 min at 2°C to 4°C to obtain a supernatant containing glutathione. The sample was then stored at −80°C until further use. The assay used for measurement of total glutathione and GSSG is based on the reaction of reduced glutathione (GSH; directly or through conversion of GSSG to GSH by 2-vinylpyridine) with 5,5′-dithio-bis (2-nitrobenzoic acid) (DTNB), which produces the 5-thio-2-nitrobenzene (TNB) chromophore measured at 412 nm. The rate of TNB formation (change in absorbance min^−1^) is proportional to the concentration of GSH in the sample. The assay was performed in 96 well plates (96 Well Tissue Culture Testplate; Spl Life Sciences, Gyeonggi-do, Korea) using an Absorbance Microplate Reader SpectraMAX 190 (Molecular Devices, Sunnyvale, CA, USA). The GSH concentration in each sample was determined by linear regression using a standardized GSH curve. The GSH/GSSG ratio was then calculated.

### Statistical analyses

Statistical significance for measurements of cores in WT and CASQ1-null mice was evaluated using a two-tailed Fisher exact test. Two-tailed Student’s unpaired *t* test or one-way ANOVA test was used for statistical analysis of all other experiments as appropriate. In all cases, statistical significance was set at either *P* < 0.05 (*) or *P* < 0.01 (**). Statistical analyses were performed using GraphPad Prism 5 (GraphPad Software, San Diego, CA, USA). All measurements were expressed as mean ± SEM.

## Results

Due to the high mortality of CASQ1-null male mice, all experiments in this study were conducted using female WT and CASQ1-null animals. See the ‘*CASQ1-null mice*’ subsection of the ‘Methods’ section and Additional file 1: Figure S1 for additional details.

### EDL muscle from CASQ1-null mice exhibit decreased force output and slowed kinetics of contraction

**Figure 1:**
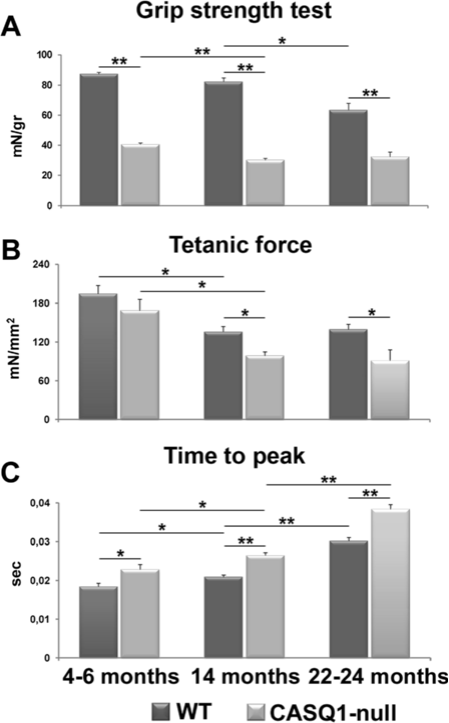
EDL muscles from CASQ1-null mice exhibit impaired force output and slowed kinetics of contraction. **(A)** Maximal grip strength (normalized to body weight) is significantly reduced across all ages in CASQ1-null mice compared to WT. **(B)** Peak-specific force measured during maximal fused isometric tetanic contractions in excised EDL muscles is significantly reduced in 14- and 22- to 24-month-old CASQ1-null mice compared to WT and in CASQ1-null mice from 4 to 6 months to 14 months of age. **(C)** Time-to-peak isometric twitch force is significantly increased in CASQ1-null mice across all ages compared to WT and increases both in WT and CASQ1-null mice up to 22 to 24 months. Number of animals tested for grip strength test: WT, *n* = 175, 31, and 9 for 4 to 6, 14, and 22 to 24 months of age, respectively; CASQ1-null, *n* = 157, 51, and 9 for 4 to 6, 14, and 22 to 24 months of age, respectively. Number of animals tested for force and contraction kinetics: WT, *n* = 5, 8, and 10 for 4 to 6, 14, and 22 to 24 months of age, respectively; CASQ1-null, *n* = 5, 5, and 6 for 4 to 6, 14, and 22 to 24 months of age, respectively. Data are given as means ± SEM (*P < 0.05, **P < 0.01). EDL, extensor digitorum brevis; CASQ1, skeletal isoform of calsequestrin; WT, wild type.

To determine the functional output of muscle from CASQ1-null mice at different ages, we performed grip strength measurements. CASQ1-null mice exhibited significantly reduced normalized grip strength (mN/g) compared to that of WT mice across all ages tested (Figure 1A), consistent with impaired neuromuscular function.

Interestingly, while grip strength in CASQ1-null mice decreased significantly between 4 to 6 and 14 months, the deficit remained stable from 14 months to 22 to 24 months. The central role of skeletal muscle in this decrease in force output was confirmed by *ex vivo* experiments in isolated EDL muscles (Figure 1B,C). Specifically, maximal tetanic-specific force (measured at optimal length at 120 Hz under isometric conditions) was significantly (*P* < 0.05) reduced in CASQ1-null mice compared to WT mice at both 14 and 22 to 24 months of age, but not at 4 to 6 months of age (Figure 1B). Again, as in the grip strength test above, peak tetanic force significantly (*P* < 0.05) decreased in CASQ1-null mice from 4 to 6 months to 14 months but remained unchanged from 14 months to 22 to 24 months. Finally, time-to-peak tetanic contraction was also significantly increased in EDL muscles from CASQ1-null mice compared to WT mice across all evaluated ages (Figure 1C; *P* < 0.05 and *P* < 0.01). Time to peak in CASQ1-null EDLs becomes progressively slower up to 22 to 24 months.

### Formation of cores in EDL muscle fibers of CASQ1-null mice

**Figure 2:**
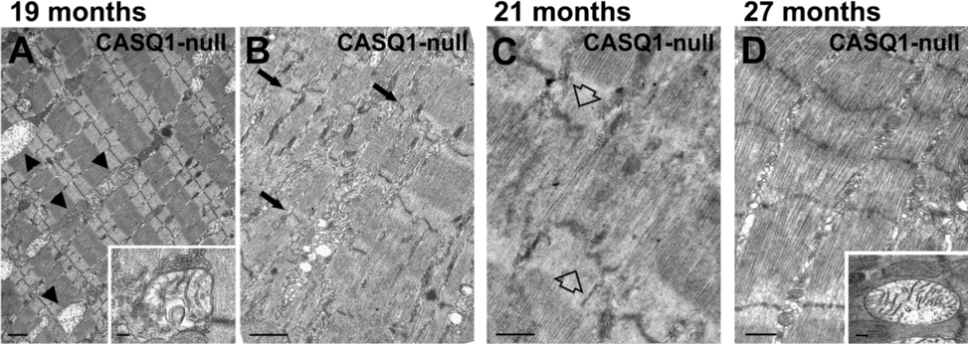
Structural abnormalities in EDL muscle fibers from aged CASQ1-null mice. **(A)** Many fibers (even at 19 months of age) do not present severe disarrangement of contractile elements. Nevertheless, even in these fibers, normal muscle ultrastructure is not well preserved, with partial misalignment of myofibrils and frequent mitochondria exhibiting clear signs of structural damage/swelling (arrowheads, see also inset). **(B, C)** *Unstructured cores*, areas in which Z lines (**(B)**, small arrows) and contractile elements are out of register and sarcomeres are partially disarranged, are frequently observed in fibers from EDL muscle from CASQ1-null mice. Z-line streaming is also present in some fibers (**(C)**, empty arrows). **(D)** *Contracture cores*, areas of excessive sarcomere shortening, contain fewer and severely damaged mitochondria (inset). See Figure 1 and Additional file 3: Table S1 for detailed quantitative analyses. Scale bars: **(A**, **B)**, 1 μm; **(C**, **D)**, 0.5 μm; insets, 0.1 μm. EDL, extensor digitorum brevis; CASQ1, skeletal isoform of calsequestrin.

**Figure 3:**
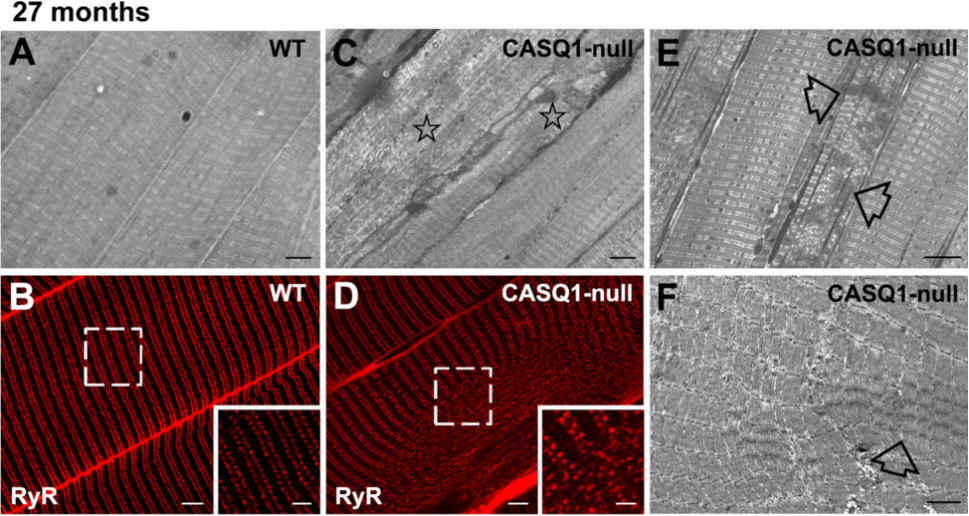
Unstructured and contracture cores in EDL muscle fibers from aged CASQ1-null mice. **(A**, **C)** Histological examination by light microscopy of EDL muscle fibers at 27 months of age. While fibers from WT mice maintain a well-preserved cross-striation pattern **(A)**, fibers from CASQ1-null mice often exhibit regions of degeneration (**(C)**, stars) classified as *unstructured cores*. **(B**, **D)** Immuno-fluorescence imaging with anti-RYR1 antibody shows loss of the precise double-row cross-striations (visible in WT, inset in **(B)** in fibers from CASQ1-null mice (inset in **(D)**). **(E**, **F)** Both histology **(E)** and electron microscopy **(F)** revealed the presence in some fibers of confined areas of contracture (empty arrows), defined as *contracture cores*, which are frequent in fibers from CASQ1-null mice. See Figure 1 and Additional file 2: Table S1 for detailed quantitative analyses. Scale bars: **(A**-**E)**, 5 μm; **(F)**, 1 μm; insets, 2 μm. EDL, extensor digitorum brevis; CASQ1, skeletal isoform of calsequestrin; RYR, ryanodine receptor; WT, wild type.

**Figure 4:**
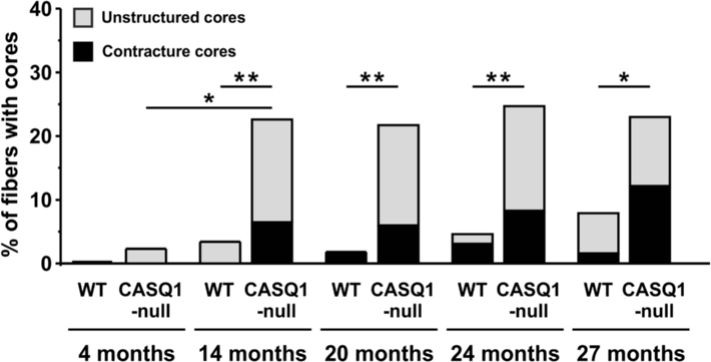
Increased incidence of unstructured and contracture cores in 14- to 27-month-old CASQ1-null mice. While the percentage of fibers in WT mice exhibiting structural cores remained low across all ages (maximum of 8% at 27 months of age), muscle fibers from CASQ1-null mice exhibited a significantly increased frequency (*P < 0.05) of structural cores from 4 months of age (approximately 2%) to 14 months (23%), remaining approximately unchanged thereafter (see also Additional file 2: Table S1 for additional detail). CASQ1, skeletal isoform of calsequestrin; WT, wild type.

To provide insight into the reduced contractility of EDL muscles from CASQ1-null mice (Figure 1), we used a combination of light and electron microscopy (EM) to compare the ultrastructure of muscle fibers of EDL muscles from age-matched WT and CASQ1-null mice. While significant structural modifications were rare at 4 to 6 months of age, the percentage of EDL fibers from CASQ1-null mice exhibiting extended areas of morphological alterations significantly increased from 4 to 6 months to 14 months and older (Figures 2, 3, and 4 and Additional file 2: Table S1). At the EM level, the structural alterations observed in fibers from CASQ1-null mice reflected a progression from modest to more severe irregularities, often characterized by the presence of swollen mitochondria (Figure 2A, arrowheads and inset) and misalignment of contractile elements (Figure 2B, arrows), some Z-line streaming (Figure 2C, empty arrows), and formation of regions of myofibril contractures (Figure 2D). Similar areas of disrupted cross-striations and/or hyper-contracture were only very rarely observed in EDL fibers of WT mice (Additional file 3: Table S1), even at the most advanced age investigated (27 months).

Although structural alterations observed in muscle fibers from CASQ1-null mice were variable in appearance, we classified two primary forms of disarray detectable by both light and electron microscopy: 1) extensive areas lacking regular cross-striation and order, termed *unstructured cores* (Figure 2B,C; and Figure 3C, stars), and 2) areas exhibiting extreme sarcomere shortening, termed *contracture cores* (Figure 2D; and Figure 3E,F, empty arrows). This terminology was previously introduced by Boncompagni *et al*. [35] in a mouse model carrying a mutation in RYR1 that results in MH with cores in humans. Such alterations were also visible by immuno-labeling with anti-RYR1 antibodies, which revealed a partial disruption in the normally highly ordered double-row pattern of CRU distribution within regions of unstructured cores (compare Figure 3B,D, white boxes and insets).

We quantified the percentage of fibers presenting either *unstructured* or *contracture cores* (Figure 4 and Additional file 3: Table S1). While fibers with extended structural alterations in CASQ1-null mice were found in only 2% of fibers at 4 months of age, their frequency increased significantly from 4 to 14 months of age (about 25%), with no further significant changes at older ages (Figure 4 and Additional file 2: Table S1, column C). However, these regions typically occupied only a limited area at early ages (14 months old), becoming much more extensive at older ages (20 to 27 months old). Fibers presenting cores were very rare in WT muscle even at the oldest age analyzed (only 8% at 27 months of age), and areas of alteration were usually very confined. Statistical analyses (Figure 4 and Additional file 2: Table S1) indicate that the total percentage of fibers presenting alterations in EDL muscles of CASQ1-null mice was significantly higher than that observed in WT mice at each of the time points analyzed except at 4 months (Figure 4).

### Reduced CSA of EDL muscle fibers from CASQ1-null mice

**Figure 5:**
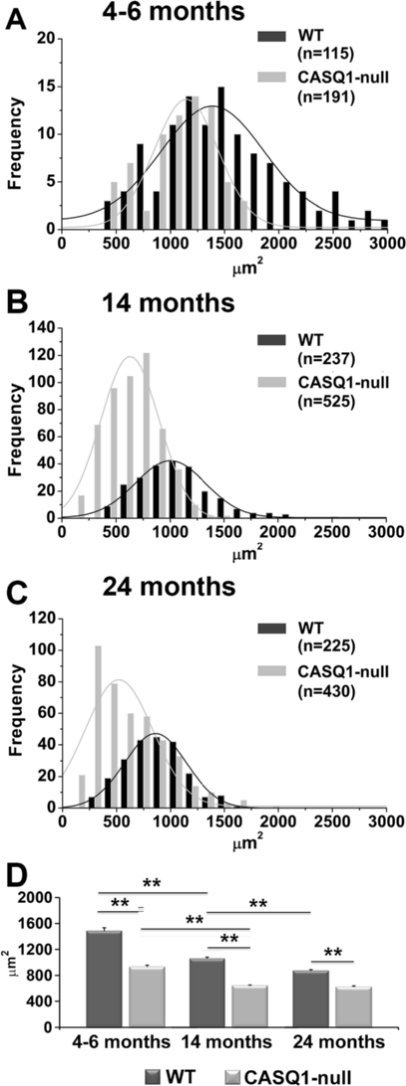
Age-dependant decrease in cross-sectional area (CSA) of EDL fibers. Frequency distributions **(A-C)** and average CSA **(D)** of individual EDL fibers at three different ages. Average fiber CSA is significantly reduced in CASQ1-null mice compared to age-matched WT mice across all age groups tested. Note that CSA of fibers decreases with age in both genotypes **(D)**, but the major reduction in fibers from CASQ1-null mice occurs from 4 to 6 months to 14 months of age, whereas this reduction occurs later in WT mice (that is, between 14 and 24 months). Data given in μm^2^ are expressed as means ± SEM (**P < 0.01). EDL, extensor digitorum brevis; CASQ1, skeletal isoform of calsequestrin; WT, wild type.

Female CASQ1-null mice exhibited a significant reduction (approximately 15%) in body weight across all ages compared to age-matched WT female mice (Additional file 3: Table S2), similar to that reported previously for adult male mice [25]. As average EDL dry weight and muscle/body weight ratio were reduced approximately 20% to 30% in CASQ1-null mice across all ages (Additional file 3: Table S2), the decrease in total body weight is likely, or at least in part, due to the reduction in muscle mass. Indeed, measurements of CSA of EDL muscle fibers at three different ages (Figure 5) verified that fiber size was significantly reduced in EDL muscles from CASQ1-null mice compared to the age-matched WT mice across all ages (*P* < 0.01). Average CSA decreased from 4 to 6 months to 24 months of age in both genotypes (33.3% in CASQ1-null mice *vs*. 41.3% of WT fibers). Interestingly, the major drop in CSA in fibers from CASQ1-null mice occurred between 4 and 14 months of age, with no further significant change observed from 14 to 24 months (Figure 5), a finding that parallels the functional (Figure 1A,B) and structural (Figure 4 and Additional file 2: Table S1) data. Microarray analyses of EDL muscles from 4-month-old CASQ1-null mice revealed a significant up-regulation of four atrogenes (CathepsinL, Bnip3, Psmd1, and Atrogin1) that belong to the autophagy and ubiquitin-proteasome pathways, suggesting one possible mechanism for the increased atrophy observed in muscle fibers from CASQ1-null mice (Additional file 4: Table S3).

### Age-dependent increase in mitochondrial damage in EDL muscle from CASQ1-null mice

**Figure 6:**
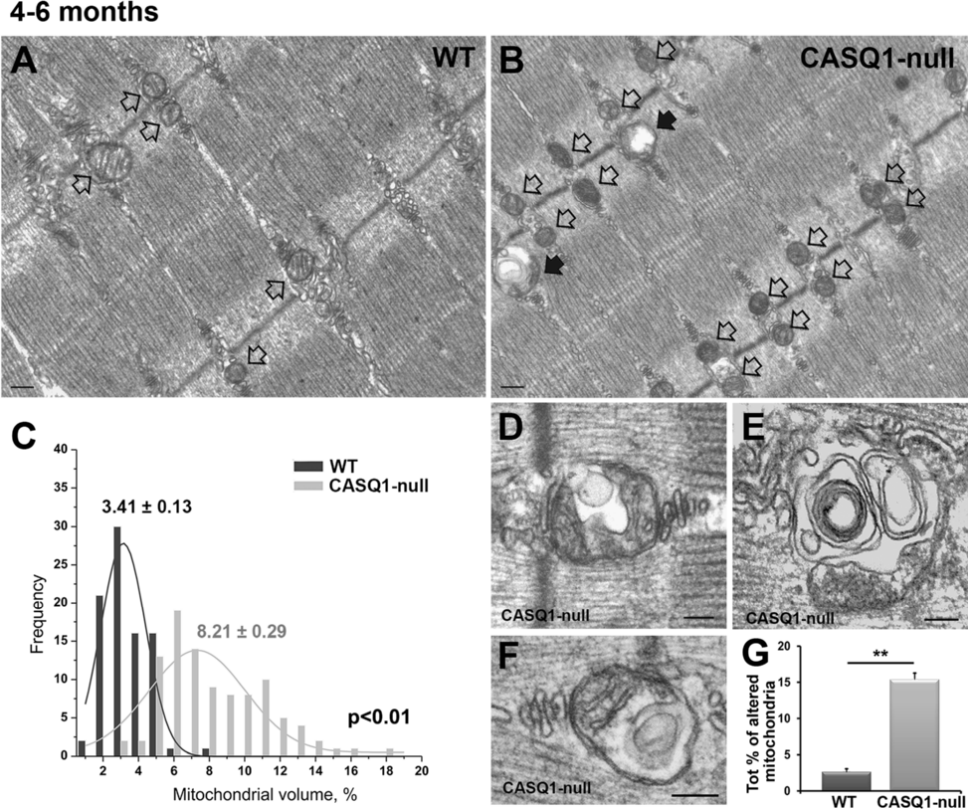
Increased mitochondrial volume and mitochondrial damage in EDL muscles from 4- to 6-month-old CASQ1-null mice. **(A, B)** Representative EM images showing increased mitochondrial number (empty arrows) in fibers from 4- to 6-month-old CASQ1-null mice **(B)** compared to WT mice **(A)** (see also ref. [25]). **(C)** Frequency distribution of the relative percentage of fiber volume occupied by mitochondria in WT and CASQ1-null fibers. Average (±SEM) percent mitochondrial volume is shown above each distribution (*P* < 0.01). **(D-G)** Representative mitochondria presenting structural abnormalities **(D**-**F)**, which are more frequent in fibers from CASQ1-null mice compared to that of WT mice **(G)**. Number of WT and CASQ1-null animals tested: *n* = 3. Number of fibers analyzed: *n* = 5. Data given as percentage ± SEM (**P < 0.01). Scale bars, **(A**, **B)**: 0.2 μm; **(D**, **E)** and **(G)**: 0.1 μm.EDL, extensor digitorum brevis; EM, electron microscopy CASQ1, skeletal isoform of calsequestrin; WT, wild type.

Muscle fibers from young (4- to 6-month-old) CASQ1-null mice do not exhibit structural cores (though clearly visible at later stages (see Figures 2, 3, and 4 and Additional file 2: Table S1). However, structural analyses at higher magnification with EM of muscle fibers at this age revealed that a) the frequency of mitochondria in muscle fibers from CASQ1-null mice is increased (Figure 6A,B, empty arrows), an observation consistent with previous findings [25], and b) mitochondria exhibiting evidence of structural damage were more frequently observed in muscle fibers from CASQ1-null mice (Figure 6B, black arrows and Figure 6G).

We measured relative mitochondrial volume (expressed as a percentage of total fiber volume) in EDL muscle from 4- to 6-month-old mice and confirmed a significant increase (approximately 2.4-fold) in cellular mitochondrial volume in muscle fibers from 4- to 6-month-old CASQ1-null mice compared to age-matched WT mice (Figure 6C; *P* < 0.01). As the transcriptional co-activator PGC-1α regulates many aspects of oxidative metabolism [44], including mitochondrial biogenesis and fiber-type switching from glycolytic toward more oxidative fibers [45], we quantified PGC-1α expression by Western blot analysis in EDL muscle homogenates from 4-month-old WT and CASQ1-null mice (Additional file 5: Figure S2). PGC-1α levels (Additional file 5: Figure S2, panel A, lower band pointed by the arrow) were increased almost twofold in EDL muscle homogenates from adult CASQ1-null mice (*P* < 0.01). This significant increase in PGC-1α suggests that the observed increase in mitochondrial volume in muscle from CASQ1-null mice is due in part from increased mitochondrial biogenesis. In addition, consistent with increased oxidative fiber content (including increased capillarization, as well as mitochondrial, myoglobin, and heme content), EDL muscles from CASQ1-null mice appear more red-colored than EDL muscles from WT mice (see Additional file 6: Figure S3).

We also quantified the number of mitochondria exhibiting one or more of the following alterations: a) swelling, b) presence of vacuoles, c) clear disruption of the external membrane and/or internal cristae, and d) myelin figures (see examples in Figure 6D,E,F). Although the frequency of damaged mitochondria varied significantly from fiber to fiber and from area to area, the percentage was significantly (*P* < 0.01) increased in muscle fibers from 4- 6-month-old CASQ1-null mice compared to that of age-matched WT mice (15.08% ± 0.9% *vs*. 2.65% ± 0.5%, respectively; Figure 6G and Table 1). Interestingly, PGC-1α expression is slightly down-regulated in CASQ1-null muscle at 25 months of age (see Additional file 5: Figure S2), suggesting that the replacement of damaged mitochondria in the muscle of aged CASQ1-null mice could be impaired, thus resulting in an accumulation of damaged mitochondria.

**Table 1:** **Percentage of mitochondria exhibiting different types of structural abnormalities in WT and CASQ1-null animals**

**EDL (4 to 6 months)**	**Mitochondrial alterations (%)**				**Total % of altered mitochondria**
	**Swollen mitochondria**	**Containing vacuole**	**Disruption of ext. membr./int. cristae**	**Containing myelin figures**	
*WT*	1.45	0.80	0.24	0.16	2.65 ± 0.5
*CASQ1-null*	6.22**	5.66**	1.96**	1.23**	15.08 ± 0.9**

In EDL muscles from 4- to 6-month-old mice, the percentage of mitochondria exhibiting one or more types of structural abnormality (swollen, disrupted internal cristae, vacuoles, or myelin figures) is significantly increased in CASQ1-null mice. Data are means ± SEM (**P < 0.01). CASQ1, skeletal isoform of calsequestrin; WT, wild type.

As detailed above, muscle fibers from CASQ1-null mice exhibited an age-dependent increase in the development of structural cores (Figures 2, 3, 4 and Additional file 2: Table S1). In order to correlate mitochondrial damage to core formation, we measured the relative volume occupied by mitochondria in non-core and core regions of aged (27 months old) CASQ1-null mice. In regions of muscle fibers exhibiting only modest alterations (similar-normal areas), mitochondrial volume was increased in aged mice compared to young adult mice (13.26% ± 0.86% *vs*. 8.21% ± 0.29% at 27 months and 4 to 6 months, respectively; Figure 6C), consistent with the visual impression of increased mitochondrial size with age (Figure 2A). Interestingly, mitochondrial volume was significantly reduced (6.01% ± 0.52%, *P* < 0.01) within unstructured and contracture cores (areas such as those in Figure 2B,C,D), consistent with loss of mitochondria in core regions.

### Increased oxidative stress and mSOF activity in muscle from CASQ1-null mice

**Figure 7:**
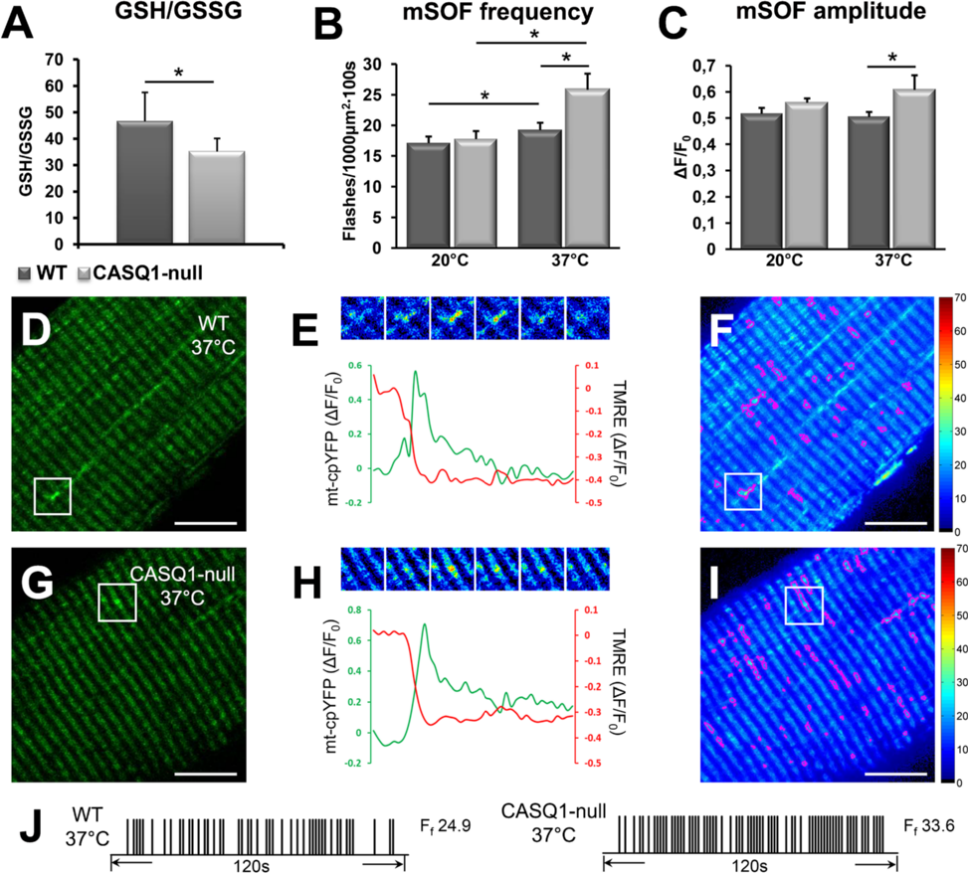
Increased oxidative stress and temperature-dependent mSOF production in muscle from CASQ1-null mice. **(A)** Compared to WT mice, muscle samples from CASQ1-null mice exhibit reduced GSH/GSSG ratio **(A)** (*n* = 6 per group). **(B, C)** Mean (±SEM *n* = 16 to 54 fibers) mSOF frequency **(B)** and amplitude **(C)** at 20°C and 37°C for FDB fibers. **(D)** Representative mt-cpYFP confocal image of an FDB fiber at 37°C from a WT mouse; boxed region indicates an area containing a mSOF. **(E)** Top: series of pseudocolor time-lapse images from the boxed region in **(D)**; bottom, time course of mt-cpYFP (green) and TMRE (red) fluorescence for the event indicated in **(D)**. **(F)** Output standard deviation map (100 images, 1.2 s/frame) of mSOF activity for the WT FDB fiber shown in **(E)**. Events are circled in magenta. **(G-I)** Same as **(D**-**F)** except for a FDB fiber at 37°C from a CASQ1-null mouse. **(J)** Representative mSOF event registry over time for the WT fiber (left) and the CASQ1-null fiber (right). Flash frequency (*F*_f_) is indicated to the right of the corresponding registry trace. Data expressed as mean ± SEM. (*P < 0.05). Scale bars: **(D**, **F**, **G**, and **I)**, 10 μm. CASQ1, skeletal isoform of calsequestrin; GSH/GSSG, reduced and oxidized glutathione; mSOF, mitochondrial superoxide flashes; mt-cpYFP, mitochondria-targeted circularly permuted yellow fluorescent protein; TMRE, tetramethylrhodamine ethyl ester; WT, wild type.

The delicate balance between the production of oxidative species and antioxidant defense is severely compromised in several pathological conditions [46]. Given that mitochondria are a primary source of reactive oxygen species (ROS) generation and mitochondrial number/volume are increased in muscle fibers of CASQ1-null mice (Figure 6), we compared the overall redox state (GSH and GSSG) and the temperature dependence of quantal mitochondrial superoxide production (termed mitochondrial superoxide flashes or mSOFs [42,47]) in skeletal muscle from 4- to 6-month-old WT and CASQ1-null mice (Figure 7). This age was chosen for these analyses because it represents a time at which mitochondrial changes have already occurred but is prior to the development of unstructured and contraction cores. The GSH/GSSG ratio was significantly (*P* < 0.01) reduced in EDL muscle homogenates from CASQ1-null mice compared to that of WT mice (Figure 7A), consistent with increased levels of oxidative stress in muscle of CASQ1-null mice. Second, we determined the frequency and amplitude (Figure 7B,C) of transient bursts of mitochondrial superoxide production that occur simultaneously with mitochondrial depolarization in quiescent FDB fibers from 4- to 5-month-old WT (Figure 7D,E,F) and CASQ1-null mice (Figure 7G,H,I). Whereas no significant differences in mSOF frequency or amplitude were detected at room temperature (20°C), a significant (*P* < 0.05) increase in mSOF frequency was observed at physiological temperature (37°C) in FDB fibers from CASQ1-null mice (17.7 ± 1.3 and 25.7 ± 1.3 flashes/1,000 μm^2^∙100 s at 20°C and 37°C, respectively) (Figure 7B,C). This temperature-dependent increase was not observed in FDB fibers from WT mice: 17.1 ± 1.1 and 19.2 ± 1.2 flashes/1,000 μm^2^∙100 s at 20°C and 37°C, respectively. Importantly, the significant (*P* < 0.05) increase in both mSOF frequency (Figure 7B) and amplitude (Figure 7C) at 37°C in fibers from CASQ1-null mice is consistent with the increased oxidative stress observed in hind limb muscles from CASQ1-deficient mice (Figure 7A). As elevated levels of ROS can damage critical cellular components including proteins and membrane lipids [48], these results may explain the increased mitochondrial damage observed in muscle fibers from 4- to 6-month-old CASQ1-null mice (Figure 6G).

### NAC treatment of CASQ1-null mice reduces oxidative stress and mSOF production, prevents mitochondrial volume/damage, and improves muscle function

**Figure 8:**
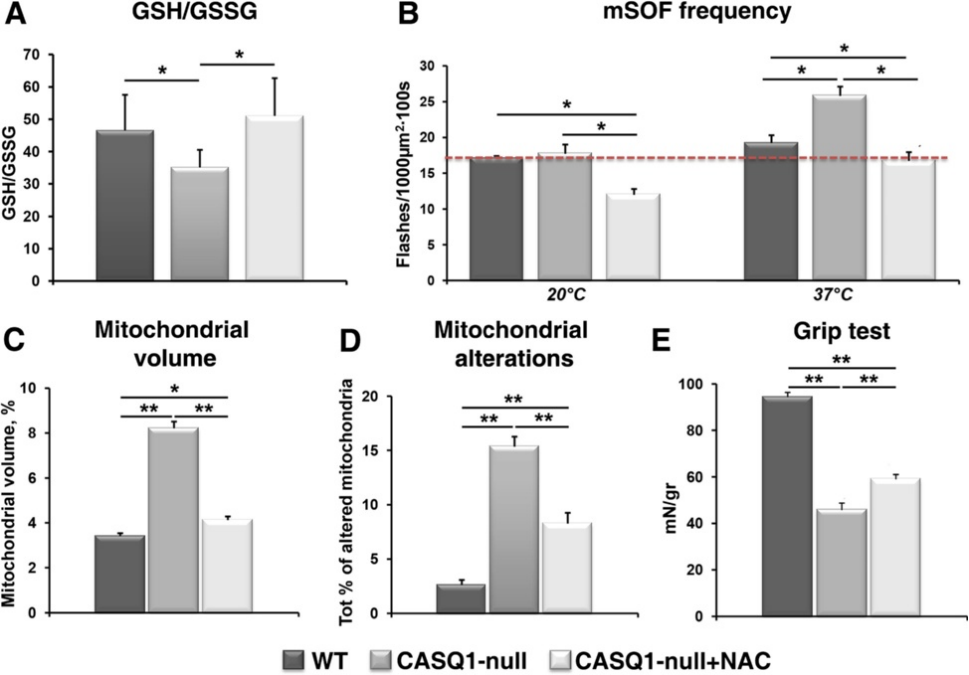
N-acetylcysteine (NAC) reduces oxidative stress, mSOF production, and mitochondrial volume/damage and improves muscle function. **(A)** GSH/GSSG ratio was restored to normal levels in muscle homogenates from CASQ1-null mice treated for 2 months with NAC (*n* = 6 per group). **(B)** Acute NAC treatment in FDB fibers reduced mSOF frequency at both 20°C and 37°C. **(C-E)** NAC treatment of CASQ1-null mice for 2 months reduced total mitochondrial volume **(C)** and mitochondrial damage **(D)** and improved grip strength **(E)**. Number of WT animals (without NAC treatment) and of old CASQ1-null animals tested: *n* = 3. Number of fibers analyzed: *n* = 5. Number of adult CASQ1-null animals (after NAC treatment) tested: *n* = 3. Number of analyzed fibers: *n* = 5. Data expressed as mean ± SEM (*P < 0.05 and **P < 0.01). CASQ1, skeletal isoform of calsequestrin; GSH/GSSG, reduced and oxidized glutathione; FDB, flexor digitorum brevis; mSOF, mitochondrial superoxide flashes; WT, wild type.

To determine if excessive oxidative stress in CASQ1-null mice is responsible for mitochondrial alterations (Figure 6), muscle damage (Figures 2, 3, and 4), and reduced contractility (Figure 1), mice were provided NAC (a potent anti-oxidant and precursor for glutathione synthesis) in their drinking water for 2 months (that is, from 2 to 4 months of age). Long-term NAC treatment *in-vivo* normalized muscle GSH/GSSG to control levels (Figure 8A; *P* < 0.05). In addition, acute *in-vitro* treatment of isolated FDB fibers with 2.5 mM NAC for 30 min reduced mSOF frequency at 20°C and 37°C in both WT and CASQ1-null mice. Specifically, NAC treatment normalized mSOF frequency at 37°C in fibers from CASQ1-null mice to a level not different to that of fibers from WT mice in the absence of NAC treatment (Figure 8B, dash line; *P* < 0.05). No change in mSOF amplitude was detected (see Additional file 7: Figure S4). Together, results in Figure 8A,B indicate that NAC treatment restored the oxidative balance muscle from CASQ1-null mice. Importantly, long-term NAC treatment of CASQ1-null mice markedly reduced structural alterations typically observed at 4 months of age, including increased mitochondrial volume (Figure 8C; *P* < 0.05 and *P* < 0.01) and mitochondria damage (Figure 8D; *P* < 0.01). Finally, NAC treatment also partially rescued overall neuromuscular function, as shown by a significant improvement in grip strength (Figure 8E; *P* < 0.01).

## Discussion

In humans, CCD and MHS are related disorders as some patients with CCD also test positive for MHS [10,16]. Thus, individuals with CCD are typically considered at risk for an MH episode during anesthesia using triggering agents (for example, halothane). In this regard, we previously reported that CASQ1-null mice are susceptible to lethal halothane- and heat-induced hypermetabolic episodes [29,31], a phenotype that closely resembles MHS in humans and mouse models expressing human RYR1 mutations [36,49].

The results presented in this study reinforce the concept that the pathophysiology of MH and CCD share a common underlying mechanism. Specifically, we demonstrate that CASQ1-null mice develop a myopathy characterized by mitochondrial damage at early ages that precede the development of structural and contracture cores at later ages (Figures 2, 3, and 4 and Additional file 2: Table S1). Similar alterations were described previously in biopsies from patients diagnosed with CCD and in RYR1 knock-in mouse models of core myopathy [35,50]. We also investigated the mechanisms that contribute to the mitochondrial damage observed in CASQ1-null mice and identified excessive production of ROS as a key early event in the myopathic cascade (possibly due to increased mitochondrial content and damage, see the ‘CASQ1-null muscle shift toward a more oxidative phenotype’ section below), confirming previous findings in a different mouse model of CCD [35,49]. The common molecular mechanism in these mouse models could be excessive SR leak (see the ‘Proposed pathogenic mechanism for mitochondrial damage and myopathy in CASQ1-null mice’ section below), resulting either directly by the mutations in the SR Ca^2+^ release channel (in RYR1^Y522S/WT^ knock-in mice) [36,49] or indirectly by the loss of CASQ1-mediated inhibition of RYR1 channel activity in CASQ1-null mice [29,31]. Importantly, we demonstrated a central role for increased oxidative stress in the mitochondrial damage and myopathy by rescuing multiple phenotypes in CASQ1-null mice with anti-oxidant treatment (Figure 8). Whereas oxidative stress, mitochondrial damage, or core formation may definitely contribute to reduce muscle strength reported in Figure 1, we cannot rule out the possibility that the impaired force output observed in CASQ1 KO muscle is still primarily due to the altered SR Ca^2+^ buffer and content and reduced Ca^2+^ release from RYRs [25-28].

### CASQ1-null muscle shift toward a more oxidative phenotype

Whereas fiber-type switching (that is, change of myosin isoform expression) does not occur in CASQ1-null mice [25], the increase in mitochondrial number [25] and volume (Figure 6) in EDL muscle of CASQ1-null mice is consistent with a shift toward increased oxidative metabolism. Consistent with this, EDL muscles from CASQ1-null mice are more red-colored than those from age-matched WT mice (Additional file 6: Figure S3). The increased mitochondrial density observed in EDL muscle fibers (Figure 6; [18]) may be due to the observed increased in PGC-1α expression (Additional file 5: Figure S2), a transcriptional co-activator that stimulates mitochondrial biogenesis and promotes the remodeling of muscle tissue toward increased oxidative metabolism [45,51]. The exact molecular signals linking CASQ1 deficiency to an up-regulation of PGC-1α remain unclear and, thus, are worthy of further investigation. However, increases in cytoplasmic Ca^2+^ and metabolic demand, as well as ATP deficiency, are all known to enhance PGC-1α expression [52-54]. Thus, since free myoplasmic Ca^2+^ levels are increased at physiological temperature in CASQ1-null fibers [29], a Ca^2+^-mediated increase in PGC-1α expression could underlie the up-regulation of this signaling pathway.

### Increased oxidative stress in CASQ1-null mice

A significant fraction of mitochondria in muscle from CASQ1-null mice exhibit morphological abnormalities (Figures 2 and 6). This increased structural damage of mitochondria (Figure 6) could result from the combined effects of elevated myoplasmic Ca^2+^ [29] and redox imbalance. Indeed, mitochondria, which are located in proximity of CRUs [25,55], are in a position to be directly impacted by Ca^2+^ disturbances at adjacent CRUs that lack CASQ1. In addition, the decrease in GSH/GSSG ratio and increase in mSOF activity (Figure 7) in muscle from CASQ1-null mice are indicative of increased mitochondrial-derived oxidative stress. Mitochondria, which produce superoxide as a byproduct of oxygen consumption due to electron slippage from the electron transport chain during aerobic respiration [56], are a primary source of ROS production within the cell. However, our results do not preclude a potential role of additional ROS sources, including NADPH oxidase [57], to the increased oxidative stress observed in muscle from CASQ1-null mice. Nevertheless, the increase in mitochondrial number/volume (Figure 6) [29] and enhanced mitochondrial superoxide production (Figure 7) likely contribute to the augmented oxidative stress observed in muscle from CASQ1-null mice (Figure 7A). These changes could in turn lead to oxidative modifications/damage to critical cellular proteins, membrane lipids, and various organelles, including mitochondria [48]. Furthermore, damaged mitochondria may not function properly and, thus, produce additional ROS, driving a dangerous feed-forward mechanism that further exacerbates cellular damage. This vicious cycle resembles that described previously in a mouse model of MH with cores (RYR1^Y522S/WT^ mice), which also exhibits a marked temperature-dependent increase in resting myoplasmic Ca^2+^, oxidative stress [49], and mSOF activity [42].

### Rescue by NAC treatment suggests a central role of oxidative stress in the myopathic cascade

Enhanced oxidative stress plays a critical role in both MH susceptibility [36,49] and the formation of cores that lack mitochondria in RYR1^Y522S/WT^ mice [35]. A similar central role of increased oxidative stress in the mitochondrial damage and muscle dysfunction observed in CASQ1-null mice is supported by data presented in Figure 8. These studies demonstrate that treatment of CASQ1-null mice with a potent anti-oxidant (NAC) for 2 months restored the muscle redox state, reduced mitochondrial alterations, and improved muscle functionality (Figure 8). By analogy to that observed for RYR1^Y522S/WT^ mice [49], we speculate that a reduction of oxidative stress in CASQ1-null mice may reduce S-nitrosylation of RYR1 to block the deleterious feed-forward cycle of increased SR Ca^2+^ leak and ROS production. However, future experiments are needed to demonstrate a critical role for RYR1 S-nitrosylation in the mitochondrial proliferation, damage, and core myopathy observed in CASQ1-null mice.

### Proposed pathogenic mechanism for mitochondrial damage and myopathy in CASQ1-null mice

**Figure 9:**
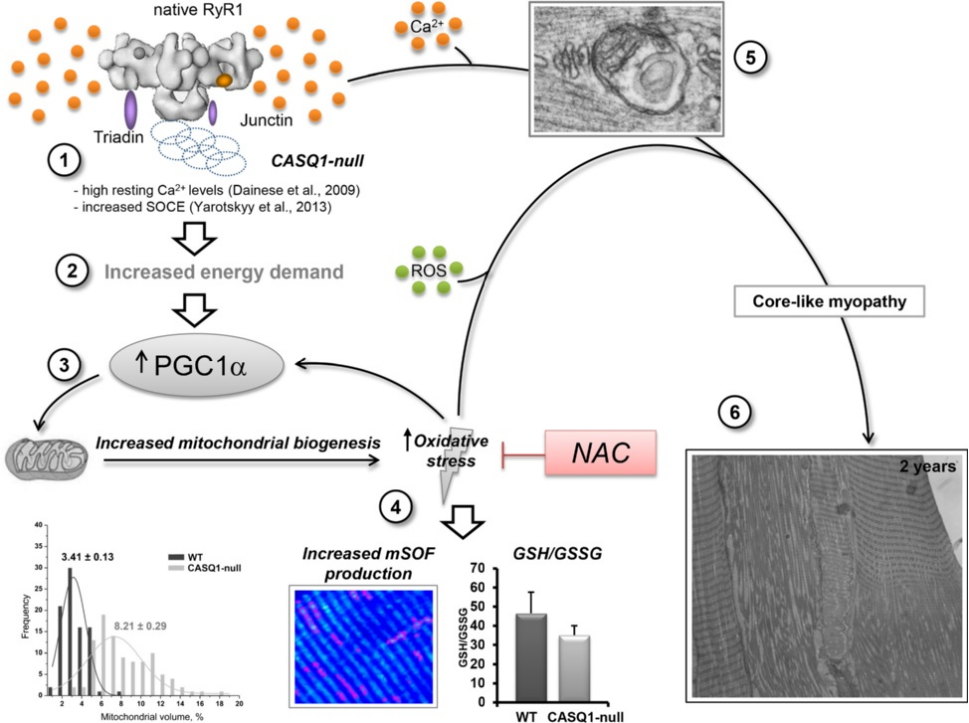
Proposed pathogenic model for myopathy in CASQ1-null mice. 1) CASQ1-null fibers exhibit elevated resting Ca^2+^ levels at body temperature [29], likely caused by increased RYR1 Ca^2+^ leak and/or enhanced SOCE [58,59]. 2) High cytoplasmic-resting Ca^2+^ increases the demand for ATP required to drive SERCA-mediated SR Ca^2+^ reuptake. 3) Increases in Ca^2+^ and energy demand enhance PGC-1α expression and, thus, mitochondrial biogenesis. 4-5) Increased metabolic rate and mitochondrial content enhance mSOF production and oxidative stress, which, over time, leads to mitochondrial damage. 6) Chronic exposure of fibers to high Ca^2+^ and oxidative stress results in formation of core-like regions (that is, myopathy). The central role of enhanced oxidative stress as a requisite step in this pathogenic model is supported by the demonstration that NAC treatment reduces mitochondrial damage and improves muscle function. Ca^2+^, calcium ions; CASQ1, skeletal isoform of calsequestrin; SR, sarcoplasmic reticulum; GSH/GSSG, reduced and oxidized glutathione; mSOF, mitochondrial superoxide flashes; PGC-1α, peroxisome proliferator-activated receptor gamma coactivator 1-alpha; RYR1, ryanodine receptor type-1; SOCE, store-operated Ca^2+^ entry; WT, wild type; NAC, N-acetylcysteine.

The schematic in Figure 9 summarizes a proposed sequence of events that leads to mitochondrial proliferation, increased oxidative stress, and mitochondrial damage and, over time, the development of a core-like myopathy in CASQ1-null mice. Dainese *et al*. [29] reported that muscle fibers from CASQ1-null mice exhibit high-resting Ca^2+^ levels at body temperature. This excess in intracellular Ca^2+^ may result from enhanced SR Ca^2+^ leak (possibly due to loss of CASQ1-mediated RYR1 inhibition) and/or excessive store depletion leading to store-operated Ca^2+^ entry (SOCE) from the extracellular space (step 1). Both effects are supported by existing literature: a) CASQ1 inhibits RYR1-opening probability and Ca^2+^ release [23,29], b) CASQ1-deficiency results in a temperature-dependent increase in resting free myoplasmic Ca^2+^ [58], and c) SOCE activation is increased in myotubes from CASQ1-null mice [58,59]. Indeed, enhanced SOCE could result from the greater susceptibility for activity-dependent SR Ca^2+^ depletion previously demonstrated in muscle fibers from CASQ1-null mice [25,27,28]. Enhanced resting Ca^2+^ levels would also increase ATP hydrolysis/demand required for SERCA-mediated SR Ca^2+^ reuptake (step 2), which together would increase PGC-1α expression [53,54] that promotes mitochondrial biogenesis and oxidative energy production (step 3). The ensuing increase in aerobic metabolism would lead to increased mitochondrial superoxide production (lowering GSH/GSSG ratio), which in turn would increase oxidative stress that further challenges mitochondrial integrity (steps 4 and 5) that promotes the formation of myopathic core-like regions (steps 6). Recent work supports a pivotal role for oxidative stress in altering the balance between protein synthesis and degradation in unloading and disuse atrophy [46].

### Differences between structural modifications in muscle in CASQ1-null mice and those described in biopsies from patients carrying the p.Asp244Gly disease mutation in CASQ1

The first mutation in the CASQ1 gene linked to human disease was recently reported [34]. In this report, a dominant missense mutation in CASQ1 (N244G) was found in a group of patients with a vacuolar myopathy characterized by weakness, fatigue, and the presence of electron-dense inclusions. The structural modifications described in biopsies from these patients do not resemble in any way the alterations (that is, unstructured and contracture cores) described in CASQ1-null mice. However, the degree to which mitochondrial alterations are observed in muscle biopsies from CASQ1 N244G vacuolar myopathy patients is currently unknown. Importantly, large SR vacuoles containing electron-dense material, which likely reflects abnormal CASQ1 aggregation, are observed in patients with the N244G CASQ1 mutation. These alterations closely resemble those described previously in skeletal muscle fibers and cardiac myocytes from mice that overexpress CASQ1 and CASQ2 [60,61]. On the other hand, in muscle fibers from CASQ1-null mice, the SR is not swollen (but is actually reduced in size), CRUs form multiple junctions with T-tubules, and the terminal cisternae of the SR lacks electron dense material (that is, CASQ1) [25].

## Conclusions

There are currently no curative treatments for CCD patients. Thus, a deeper mechanistic understanding of the molecular mechanisms that underlie mitochondrial damage and the formation of cores in CCD is needed in order to identify potential new therapeutic targets and, thus, to develop effective new interventions for this disorder. The mechanisms responsible for the loss of mitochondrial activity in core regions of muscle from CCD patients have also not yet been elucidated. Especially challenging is to explain how both gain- and loss-of-function mutations in RYR1 lead to myopathies characterized by damage and destruction of mitochondria (that is, CCD and multi-minicore disease), formation of structural and contracture cores, and muscle weakness.

Mutations in the CASQ1 gene have not been identified in patients diagnosed with either CCD or in MHS [32], and this represents a clear limit of our mouse model. Nevertheless, data of our study show that CASQ1 deficiency results in an age-dependent myopathy characterized by mitochondrial damage and formation of unstructured/contracture cores resembling those described in muscle biopsies from human CCD patients and in other mouse models of MH and CCD (see ref. [35,50]). In addition, we found that the molecular mechanisms observed in CASQ1-null fibers (that is, Ca^2+^ leak, excessive oxidative stress, mitochondrial damage) parallel those reported previously in a knock-in mouse carrying a human mutation [35].

We propose that increased oxidative stress (Figure 7), likely resulting from an imbalance of intracellular Ca^2+^ homeostasis [25,26,28-30], is a critical early myopathic trigger in CASQ1-null mice. In support of this hypothesis, we show that treatment with NAC, a potent antioxidant, corrected the increased oxidative stress, reduced the incidence of mitochondrial damage, and improved muscle function of CASQ1-null mice (Figure 8). These results suggest that NAC (or other anti-oxidants, [42,47]) could be considered as a therapeutic intervention to prevent mitochondrial damage and improve muscle function in CCD and, more generally, to mitigate the deleterious effects of increased oxidative stress and mitochondrial damage in other muscle disorders [62].

## Additional files

Additional file 1: Figure S1. Age-dependent survival curve. This is a figure showing the mortality rate of male and female WT and CASQ1-null mice. Detailed description is provided within the file. Additional file 2: Table S1. Analysis of structural disarray. Detailed description is provided within the file. Additional file 3: Table S2. Body and muscle weight. Detailed description is provided within the file. Additional file 4: Table S3. Gene microarray analysis. Detailed description is provided within the file. Additional file 5: Figure S2. PGC-1α expression. This is a figure showing representative Western blot of PGC-1α of EDL total homogenates at 4 and 25 months of age. Detailed description is provided within the file. Additional file 6: Figure S3. Dissected EDL muscles. This is a figure showing the color of EDL muscles from WT and CASQ1-null mice. Detailed description is provided within the file. Additional file 7: Figure S4. Mitochondrial superoxide flashes. This is a figure showing the temperature dependence of mitochondrial superoxide flash activity in the presence or absence of NAC. Detailed description is provided within the file.

## Abbreviations

Ca^2+^: calcium ions
CASQ1: skeletal isoform of calsequestrin
CCD: central core disease
CRU: calcium release unit
CSA: cross-sectional area
α_1s_DHPR: skeletal muscle isoform of dihydropyridine receptor
DTNB: 5,5′-dithio-bis (2-nitrobenzoic acid)
EC coupling: excitation-contraction coupling
EDL: extensor digitorum brevis
EM: electron microscopy
FDB: flexor digitorum brevis
GSH and GSSG: reduced and oxidized glutathione
MH: malignant hyperthermia
MHS: malignant hyperthermia susceptibility
mSOF: mitochondrial superoxide flashes
mt-cpYFP: mitochondria-targeted circularly permuted yellow fluorescent protein
NAC: N-acetylcysteine
O.D.: optical density
PGC-1α: peroxisome proliferator-activated receptor gamma coactivator 1-alpha
ROS: reactive oxygen species
RT: room temperature
RYR: ryanodine receptor
SOCE: store-operated Ca^2+^ entry
SR: sarcoplasmic reticulum
TMRE: tetramethylrhodamine ethyl ester
TNB: 5-thio-2-nitrobenzene
T-tubule: transverse tubule
WT: wild type
