# Supplementary material for: Oxidative stress, mitochondrial damage, and cores in muscle from calsequestrin-1 knockout mice
Source: Skelet Muscle. 2015 Apr 18;5:10. doi: 10.1186/s13395-015-0035-9 (PMC4464246; doi:10.1186/s13395-015-0035-9)
Supplement: Additional file 1: Figure S1. — Age-dependent survival curve. This is a figure showing the mortality rate of male and female WT and CASQ1-null mice. Detailed description is provided within the file. [file 13395_2015_35_MOESM1_ESM.pdf]

ADDITIONAL FILE 1

Figure S1.

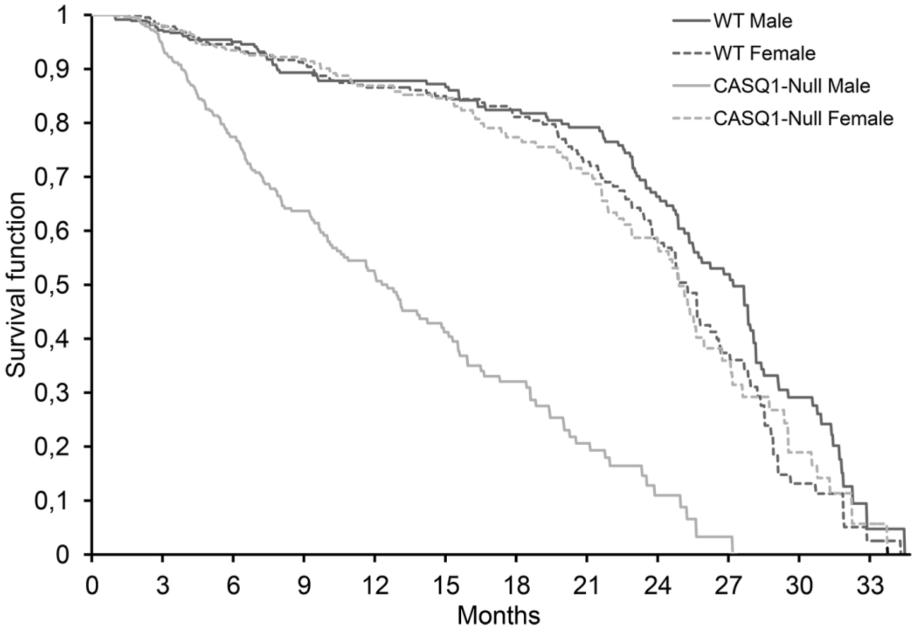

Figure S1. Age-dependent survival curves for male and female WT and CASQ1-null mice housed under standard conditions and evaluated using the Kaplan-Meier method. The mortality rate of male CASQ1-null mice was significantly higher than that of either CASQ1-null females, WT males, or WT females. Given the lack of a sufficient number of aged CASQ1-null male mice (e.g. 1 year of age), all experiments in this study were conducted in female animals.
