# Supplementary material for: Oxidative stress, mitochondrial damage, and cores in muscle from calsequestrin-1 knockout mice
Source: Skelet Muscle. 2015 Apr 18;5:10. doi: 10.1186/s13395-015-0035-9 (PMC4464246; doi:10.1186/s13395-015-0035-9)
Supplement: Additional file 2: Table S1. — Analysis of structural disarray. Detailed description is provided within the file. [file 13395_2015_35_MOESM2_ESM.pdf]

ADDITIONAL FILE 2

Figure S2.

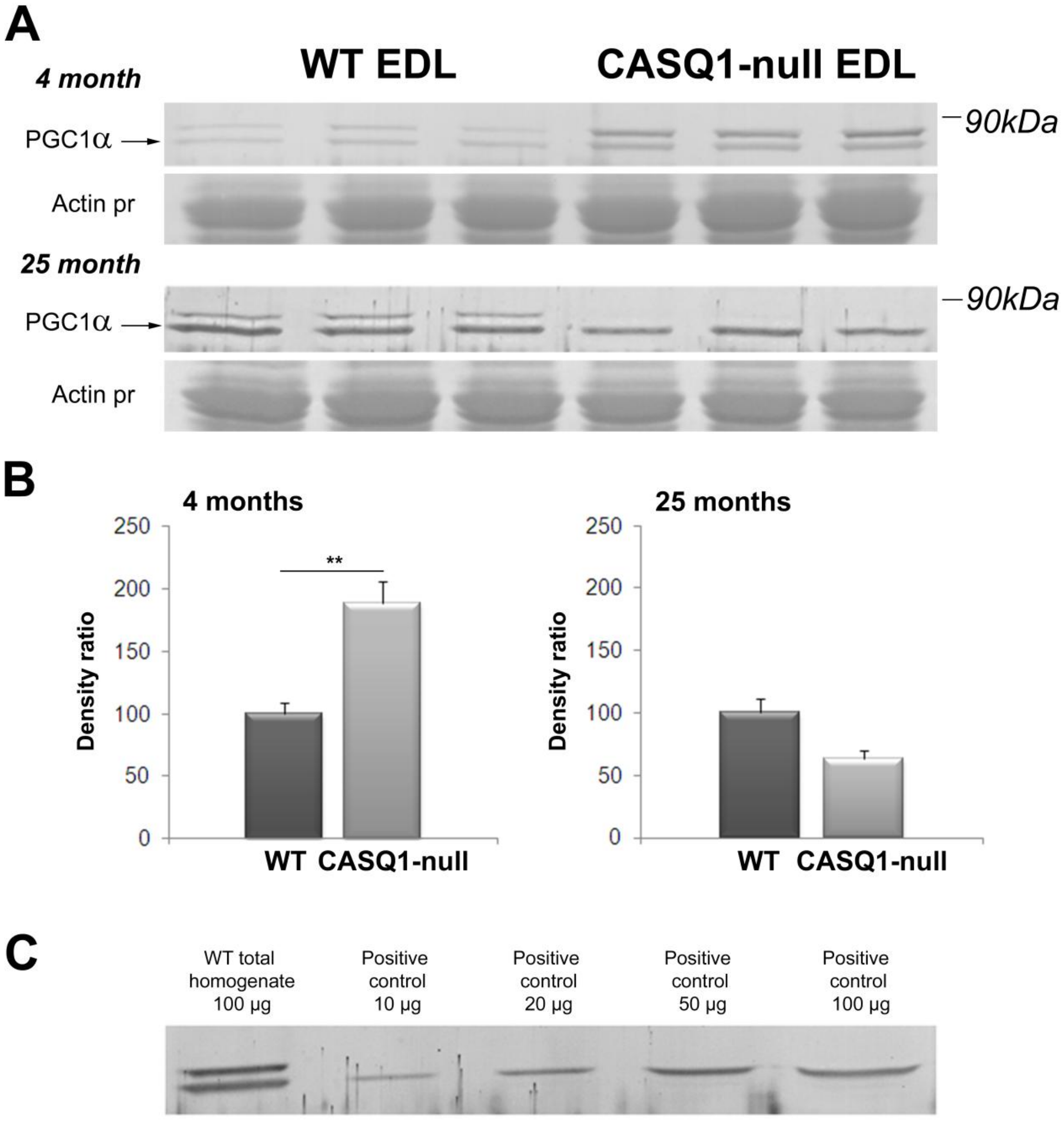

Figure S2. Over and under expression of PGC1 α in adult (4 month old) and old (25 month old) CASQ1-null EDL. A) PGC1α representative western blot and loading control (ponceau red of actin) of EDL total homogenates at 4 and 25 month old. B) Densitometric analyses show variation of PGC1α expression with age: a significant increase in adult (4 month old) and a decrease in old (25 month old) CASQ1-null EDL muscles compared to that of age- and WT mice (the latter set as 100

O.D.). C) A nuclear extract positive control was used to demonstrate that the lower band in the doublet corresponds to PGC1α. **$p<0.01$.
