# Supplementary material for: Oxidative stress, mitochondrial damage, and cores in muscle from calsequestrin-1 knockout mice
Source: Skelet Muscle. 2015 Apr 18;5:10. doi: 10.1186/s13395-015-0035-9 (PMC4464246; doi:10.1186/s13395-015-0035-9)
Supplement: Additional file 3: Table S2. — Body and muscle weight. Detailed description is provided within the file. [file 13395_2015_35_MOESM3_ESM.pdf]

ADDITIONAL FILE 3

Table S1. Quantitative analyses of fibers containing areas of structural disarray (unstructured cores and contracture cores) reveals that alterations are far more frequent in CASQ1-null fibers.

EDL ***(n=2)***	**Age (months)**	**Total No. of analyzed fibers (No. of mice)**	**No. of fiber with alterations**		**C % Total fiber area altered**
			**A Unstructured Cores**	**B Contracture Cores**	
**WT**	4	34 (2)	0 (0%)	0 (0%)	**0**
**WT**	14	58 (2)	2 (3%)	0 (0%)	**3**
**WT**	20	56 (2)	0 (0%)	1 (2%)	**2**
**WT**	24	65 (2)	1 (2%)	2 (3%)	**5**
**WT**	27	63 (2)	4 (6%)	1 (2%)	**8**
**CASQ1-null**	4	41 (2)	1 (2%)	0 (0%)	**2**[&]
**CASQ1-null**	14	62 (2)	10 (17%)	4 (6%)	**23****
**CASQ1-null**	20	83 (2)	13 (16%)	5 (6%)	**22****
**CASQ1-null**	24	85 (2)	14 (16%)	7 (8%)	**24****
**CASQ1-null**	27	81 (2)	9 (11%)	10 (13%)	**24***

Columns A and B) Fibers exhibiting unstructured and contracture cores are frequent in EDL muscles of CASQ1-null mice starting at 14 months, but rare in EDL muscles of age-matched WT mice. There it seems to be trend for an increased percentage of contracture cores (from 6 to 13% between 14 and 27 months of age), which suggests that probably unstructured cores may be an early-point in formation of contracture cores. Column C) The total percentage of fiber area exhibiting ultrastructural decay (unstructured cores + contraction cores) increases significantly from 4 to 14 months of age in CASQ1-null muscles, but remains unchanged at later stages. *p< 0.05 and **p <0.01 compared to age matched WT; [&]p < 0.05 compared to 14-month-old CASQ1-null.
