# Supplementary material for: Oxidative stress, mitochondrial damage, and cores in muscle from calsequestrin-1 knockout mice
Source: Skelet Muscle. 2015 Apr 18;5:10. doi: 10.1186/s13395-015-0035-9 (PMC4464246; doi:10.1186/s13395-015-0035-9)
Supplement: Additional file 5: Figure S2. — PGC-1α expression. This is a figure showing representative Western blot of PGC-1α of EDL total homogenates at 4 and 25 months of age. Detailed description is provided within the file. [file 13395_2015_35_MOESM5_ESM.pdf]

ADDITIONAL FILE 5

Table S3. Gene microarray analyses of EDL muscles from WT and CASQ1-null mice reveals significant up-regulation of genes involved in autophagy/atrophy signaling.

GB_accession	*Gene_Symbol*	*Description*	*Fold Change*[A]
**NM 026346**	atrogin-1/MAFbx	E3 ubiquitin ligase	1,82445753
**AK010596**	Psmd1	proteasome (prosome, macropain) 26S subunit, non-ATPase, 1	1,63895746
**X06086**	Ctsl	Cathepsin L	1,46843157
**AF041054**	Bnip	BCL2/adenovirus E1B 19 kDa-interacting protein 1, NIP3	1,61282553

Microarray analyses revealed significant up-regulation of 4 atrogenes involved in autophagy/atrophy signalling. Cathepsin L is a lysosomal enzyme involved in the degradation of membrane proteins that is upregulated during skeletal muscle atrophy [1]; Bnip3 regulates autophagy by inducing mitochondrial damage and removal via autophagosomes (mitophagy) [2]. Atrogin1/MAFbx is an ubiquitin ligase that contributes to muscle atrophy in a variety of catabolic states [3]. Psmd1 is a regulatory subunit of the proteasome that is involved in the ATP-dependent degradation of ubiquitinated proteins [4]. The up-regulation of these genes in EDL muscle from CASQ1-null mice reflects activation of the proteosomal and autophagic pathways during muscle atrophy.

SUPPLEMENTAL REFERENCES

1. Judge AR, Koncarevic A, Hunter RB, Liou HC, Jackman RW, Kandarian SC. Role for IkappaBalpha, but not c-Rel, in skeletal muscle atrophy. Am J Physiol Cell Physiol. 2007;292(1):C372-82. doi:00293.2006 [pii] 10.1152/ajpcell.00293.2006.
2. Kubli DA, Ycaza JE, Gustafsson AB. Bnip3 mediates mitochondrial dysfunction and cell death through Bax and Bak. Biochem J. 2007;405(3):407-15. doi:BJ20070319 [pii] 10.1042/BJ20070319.
3. Li YP, Chen Y, John J, Moylan J, Jin B, Mann DL et al. TNF-alpha acts via p38 MAPK to stimulate expression of the ubiquitin ligase atrogin1/MAFbx in skeletal muscle. Faseb J. 2005;19(3):362-70. doi:19/3/362 [pii] 10.1096/fj.04-2364com.
4. Voges D, Zwickl P, Baumeister W. The 26S proteasome: a molecular machine designed for controlled proteolysis. Annu Rev Biochem. 1999;68:1015-68. doi:10.1146/annurev.biochem.68.1.1015.
