# Supplementary material for: Oxidative stress, mitochondrial damage, and cores in muscle from calsequestrin-1 knockout mice
Source: Skelet Muscle. 2015 Apr 18;5:10. doi: 10.1186/s13395-015-0035-9 (PMC4464246; doi:10.1186/s13395-015-0035-9)
Supplement: Additional file 6: Figure S3. — Dissected EDL muscles. This is a figure showing the color of EDL muscles from WT and CASQ1-null mice. Detailed description is provided within the file. [file 13395_2015_35_MOESM6_ESM.pdf]

ADDITIONAL FILE 6

Figure S3.

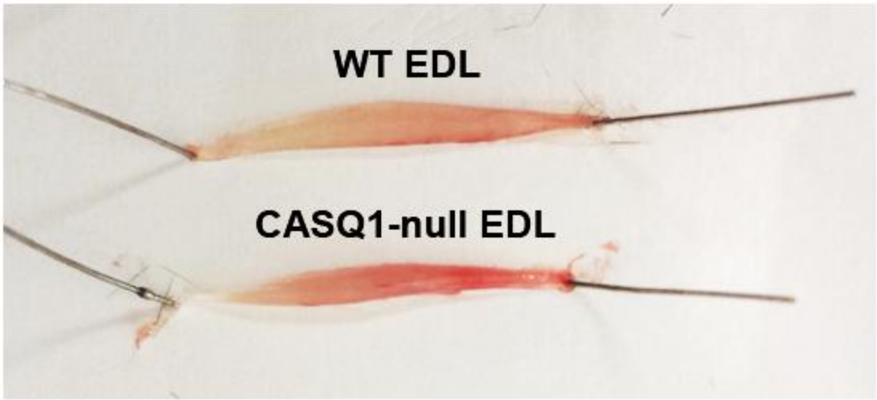

Figure S3. Dissected EDL muscles of CASQ1-null mice are more red-colored than those from WT mice. Representative EDL muscles from 4 months old WT (upper) and CASQ1-null (lower) mice reveals that EDL muscle from CASQ1-null mice are more red-colored, consistent with increased capillarization and myoglobin/heme content.
