# Supplementary material for: Oxidative stress, mitochondrial damage, and cores in muscle from calsequestrin-1 knockout mice
Source: Skelet Muscle. 2015 Apr 18;5:10. doi: 10.1186/s13395-015-0035-9 (PMC4464246; doi:10.1186/s13395-015-0035-9)
Supplement: Additional file 7: Figure S4. — Mitochondrial superoxide flashes. This is a figure showing the temperature dependence of mitochondrial superoxide flash activity in the presence or absence of NAC. Detailed description is provided within the file. [file 13395_2015_35_MOESM7_ESM.pdf]

ADDITIONAL FILE 7

Figure S4.

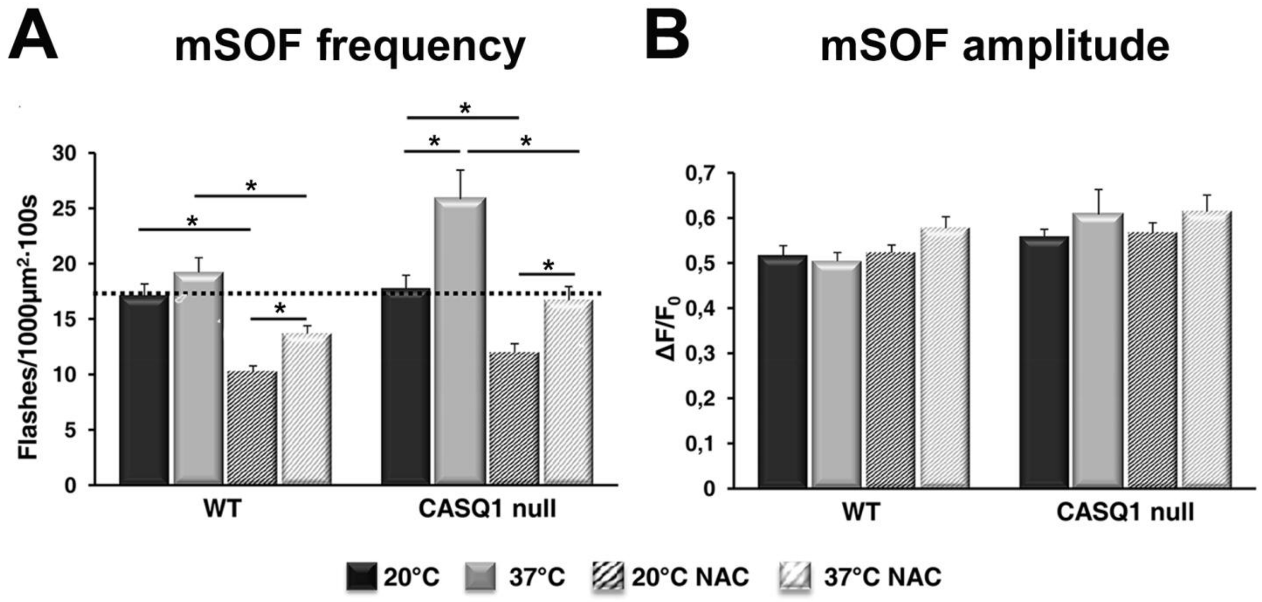

Figure S4. Temperature dependence of mitochondrial superoxide flash activity in FDB fibers from WT and CSQ1-null mice with and without NAC treatment. Average superoxide flash frequency (A) and amplitude (B) measured in FDB fibers from WT (n=21-54 cells from 2-4 female mice) and CASQ1-null mice (n=16-51 cells from 2-4 female mice) maintained at 20°C and 37°C in either the absence or presence of NAC (2.5 mM for 30 min). mSOF frequency was significantly increased at 37°C in fibers from CASQ1-null mice, but not in WT mice. NAC treatment significantly reduced mSOF frequency at both 20°C and 37°C in fibers from both WT and CASQ1-null mice. mSOF frequency of NAC-treated fibers from CASQ1-null mice at 37°C was not different that of untreated fibers from WT mice at 37°C. Data are given as mean ± SEM. ($^{*}p<0.05$).
